# Cellular Senescence in Intervertebral Disc Aging and Degeneration: Molecular Mechanisms and Potential Therapeutic Opportunities

**DOI:** 10.3390/biom13040686

**Published:** 2023-04-18

**Authors:** Prashanta Silwal, Allison M. Nguyen-Thai, Haneef Ahamed Mohammad, Yanshan Wang, Paul D. Robbins, Joon Y. Lee, Nam V. Vo

**Affiliations:** 1 Ferguson Laboratory for Spine Research, Department of Orthopaedic Surgery, University of Pittsburgh, Pittsburgh, PA 15261, USA; 2 Department of Chemistry and Biochemistry, University of California, Los Angeles, CA 90095, USA; 3 Department of Health Information Management, University of Pittsburgh, Pittsburgh, PA 15260, USA; 4 Institute of the Biology of Aging and Metabolism and Department of Biochemistry, Molecular Biology and Biophysics, University of Minnesota, Minneapolis, MN 55455, USA

**Keywords:** intervertebral disc degeneration, cellular senescence, senescent cells, aging, senotherapy, senolytics

## Abstract

Closely associated with aging and age-related disorders, cellular senescence (CS) is the inability of cells to proliferate due to accumulated unrepaired cellular damage and irreversible cell cycle arrest. Senescent cells are characterized by their senescence-associated secretory phenotype that overproduces inflammatory and catabolic factors that hamper normal tissue homeostasis. Chronic accumulation of senescent cells is thought to be associated with intervertebral disc degeneration (IDD) in an aging population. This IDD is one of the largest age-dependent chronic disorders, often associated with neurological dysfunctions such as, low back pain, radiculopathy, and myelopathy. Senescent cells (SnCs) increase in number in the aged, degenerated discs, and have a causative role in driving age-related IDD. This review summarizes current evidence supporting the role of CS on onset and progression of age-related IDD. The discussion includes molecular pathways involved in CS such as p53-p21^CIP1^, p16^INK4a^, NF-κB, and MAPK, and the potential therapeutic value of targeting these pathways. We propose several mechanisms of CS in IDD including mechanical stress, oxidative stress, genotoxic stress, nutritional deprivation, and inflammatory stress. There are still large knowledge gaps in disc CS research, an understanding of which will provide opportunities to develop therapeutic interventions to treat age-related IDD.

## 1. Introduction

With increased human lifespan, there is a rapid global rise in prevalence and impact of age-dependent diseases. Among the age-associated chronic pathological conditions, low back pain (LBP) is a major public health concern with long-term socioeconomic consequences. The etiology of LBP is multifactorial; however, degeneration of intervertebral discs (IVDs) is a main contributor. Intervertebral discs degeneration (IDD) is driven primarily by aging, fueling the disease progression. During aging, IVDs undergo structural, biochemical, biomechanical, and functional changes that can lead to LBP and other spine-related pathologies [1]. Although aging is a primary driver of IDD, IDD progression can be fueled by genetic predisposition, injury, obesity, smoking, and abnormal mechanical loading leading to molecular, cellular, and tissue changes in the IVDs.

The IVDs provide flexibility and shock-absorbing function within the spine. Each IVD consists of the inner gelatinous nucleus pulposus (NP), outer fibrous annulus fibrosus (AF), and cartilaginous endplates anchoring the disc to the adjacent vertebrae. The NP dissipates the compressive forces toward the surrounding AF and serves as shock absorber, while the AF controls the tensile forces during bending and stretching of the spine [2]. During aging, the AF loses its organized fibrous lamellae meshwork, and the NP undergoes fibrotic changes resulting from loss of proteoglycans in the extracellular matrix (ECM) and subsequent loss of water content. Aged IVDs harbor tissue fissures and disc height is reduced as a result of these changes [2]. Other pathological changes in IVDs during IDD include increased inflammation, apoptosis, and cellular senescence (CS). Loss of disc ECM is due to reduced matrix synthesis and enhanced catabolism with the rise in level of matrix metallopeptidases (MMPs), disintegrin, and a disintegrin and metalloproteinase with thrombospondin motifs (ADAMTS). A key initiator of ECM degradation during the course of IDD is chronic inflammation, which results from increased secretion of pro-inflammatory cytokines (i.e., interleukin (IL)-1β, tumor necrosis factor (TNF)-α, and IL-6). Taken together, these hallmarks of IDD lead to reduced structural integrity and biomechanical function of the spine.

## 2. Overview of Cellular Senescence

Cellular Senescence is the process of irreversible loss of cell proliferation that occurs in response to developmental signals and accumulation of unrepaired damage in deoxyribonucleic acid (DNA). It is a complex and multistep dynamic process for which knowledge is still evolving with new experimental findings. The early CS step is generally defined by cells exiting their cell cycle to enter the stable cell-cycle arrest phase through sustained activation of the p16^INK4a^ and/or p53–p21 pathways. Full CS occurs when early CS cells subsequently undergo morphological changes, chromatin remodeling and secretion of senescence-associated secretory phenotype (SASP) factors. Progression to late CS is thought to be driven by additional genetic and epigenetic changes, including chromatin budding, histone proteolysis and retrotransposition, promoting further transcriptional change and SASP heterogeneity [3,4]. There are two types of CS: (1) replicative senescence (RS) caused by telomere shortening; and (2) stress-induced premature senescence (SIPS) caused by accumulation of DNA damage and other types of cellular stress [5]. Major hallmarks of CS include cell-cycle withdrawal, macromolecular damage, SASP and deregulated metabolism, which are interdependent to each other during activation of senescence [6]. Cells with SASP produce and secrete an abundance of pro-inflammatory cytokines and chemokines, growth factors, and MMPs [6]. Many stimuli that activate senescence are stress triggers such as telomere erosion, DNA damage, lysosomal stress, oncogene activation, and oxidative stress. These stressors activate cell cycle arrest and resistance to mitogen and oncogenic transformation, leading to CS. The induction of chronic senescence and accumulation of senescent cells (SnCs) in the local microenvironment leads to tissue damage and degeneration.

To identify SnCs or the process of senescence, multiple biomarkers are used, including increased expression of cell cycle control kinases such as p53, p21^CIP1^, p16^INK4a^, and phosphorylated retinoblastoma (Rb). Other biomarkers of CS include telomere damage, increased levels of SASP factors, activity of senescence-associated beta-galactosidase (SA-β-gal), and expression of anti-apoptotic markers such as Bax-1. It is difficult to define senescence with a single biomarker due to the heterogeneity of senescent cells and lack of a single specific marker that reliably determines CS; therefore, it is important to use more than one biomarker to confirm the presence of CS.

## 3. Cellular Senescence in Aging and Degenerating Disc

**Figure 1:**
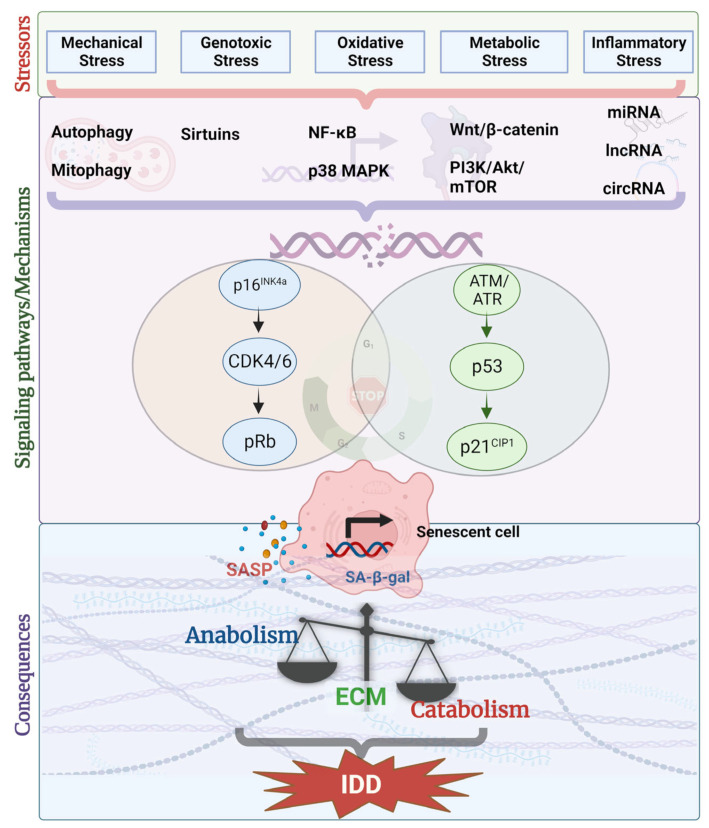
Overview of cellular senescence during intervertebral disc degeneration. The reported pathways of stressors, signaling molecules, and consequences of cellular senescence (CS) during intervertebral disc degeneration (IDD) are depicted. During aging, disc CS can be caused by mechanical, genotoxic, oxidative, metabolic, and inflammatory stress which activate signaling cascades such as NF-kB, mitogen activated protein kinases (MAPKs), Sirtuins, PI3K/Akt/mTOR, etc., and cellular mechanisms such as autophagy and mitophagy or they directly activate the DNA damage response to induce the senescence pathway. The senescence signals and DNA damage responses activate the ATM-p53-p21^CIP1^ and p16^INK4a^-pRb pathways, which work reciprocally with each other to induce cell cycle arrest and senescence. Disc CS associated secretory phenotype (SASP) produces and secretes pro-inflammatory and catabolic factors such as MMPs to further amplify the process of disc degeneration. As a consequence of changed cellular phenotype, extracellular matrix (ECM) catabolism is increased, and anabolism is reduced leading to degradation of the disc tissue. (Figure created with BioRender.com, accessed on 8 March 2023).

The number of SnCs is increased during IVD aging and degeneration in both human tissues and animal models [1,7,8,9]. Earlier studies of human disc tissue revealed an increase in the SA-β-gal positive senescent cells in herniated discs as compared to those from non-herniated discs [10]. Similarly, SA-β-gal positive cells were also identified in aging and degenerating human and sand rat discs [9]. A rise in SnCs was observed in IVDs of aged mice [1,7] sheep [11], and rabbits [12], implicating CS in the pathogenesis of aging-related IDD. Subsequently, numerous studies have reported biomarkers of CS in disc cells. In humans, p16^INK4a^ is increased in an age-dependent manner in non-degenerative discs and is also increased in cells from degenerative discs in both young and old persons [8,13]. Interestingly, in vitro studies have indicated that NP cells exposed to serial subculture, ionizing radiation, and p16^INK4a^ overexpression express similar transcript profile of many catabolic and inflammatory genes found in SASP independent of senescence triggers [14]. An overview of the reported stress triggers of IVD CS and key pathways involved is presented in Figure 1.

Although there is extensive descriptive research on disc CS, more vigorous studies are needed to elucidate the mechanisms of disc aging and degeneration in the context of senescence and its cross talk with several cellular pathways such as autophagy and inflammation. This is necessary to understand CS in order to prevent, delay, or ameliorate the progression of age-dependent IDD [2]. The present review aims to analyze the current disc CS studies to uncover the general mechanisms of senescence in IDD. Deep understanding of these mechanisms is currently absent but absolutely required for the development of effective therapeutic interventions to treat age-related IDD.

For this review, we used the natural language processing (NLP) method to extract the relevant articles from PubMed, mostly published recently in the field of CS. We used the following Boolean query in combination with Medical Subject Headings (MeSH) terms to retrieve relevant CS papers from PubMed: “Cellular Senescence”[MeSH] OR “Cellular Senescence/physiology”[MeSH]) AND (“Intervertebral Disc/metabolism”[MeSH] OR “Intervertebral Disc”[MeSH] OR “Intervertebral Disc/pathology”[MeSH]. For this review, we screened the title and abstract of each paper and selected the most relevant papers that reported disc CS studies.

## 4. Disc Cell Senescence under Various Stressors

Cell culture models have been useful tools to study the mechanisms of senescence and its impact on age-related IDD. In addition to replicative senescence, incubation of primary cells with different stressors for several days induces SIPS or acute CS as it is termed in some cases [5,15]. Hence, stimulation of disc cells with external stimuli leads to activation of SIPS, which along with in vivo IDD studies are used to study CS and IDD. In this review, wherever possible we pointed out if the study was assessing premature/acute senescence or replicative senescence for readers to identify the context behind the studies. Typically, cells grown in culture require about two weeks post exposure to genotoxic or oxidative stress to establish true senescence phenotype seen in SIPS. Although not clearly mentioned, the stimulation of disc cells with external stimuli for 1–3 days reported in many studies most likely accounts for activation of the acute response to stress and not true SIPS. More investigation is needed to differentiate between replicative senescence, SIPS, and mere acute response to stress in disc cells.

### 4.1. Mechanical Stress

Abnormal mechanical stress is a major etiological factor in the pathogenesis of IDD. Several studies have linked mechanical stress in IVD with increased CS level in the disc. Static or dynamic compression in disc organ culture induces senescence of NP cells [16]. Piezo1, a mechanosensitive ion channel, plays a significant role in linking the mechanical stress to the biological signals in IDD [17]. Piezo1 expression is significantly higher in NP tissue of IDD patients compared to healthy samples [18]. Excessive mechanical stress is responsible for Piezo1 overexpression leading to mitochondrial damage and inflammatory responses further triggering apoptosis and reducing the autophagy, which triggers CS and dysfunction [18].

In a cellular model of mechanical stress, compression of NP cells amplified the expression of the senescence markers p53 and p16^INK4a^ [19]. Mechanical stress exerted on a hydrogel culture of NP cells promoted senescence and SASP via nuclear factor kappa-light-chain-enhancer of the activated B cells (NF-κB) pathway [19]. Piezo1-mediated activation of the NF-κB pathway in NP cells under mechanical stress upregulated the expression of periostin, which is secreted by SnCs to activate NF-κB, thereby forming an activation loop to speed senescence [19]. Of note, Yoda1, a specific activator of Piezo1 induced the Ca^2+^ influx leading to activation of NF-κB and CS in human NP cells, resulting in severe IDD in rat tails in vivo [19]. Matrix stiffness also activates Piezo1 leading to increased intracellular Ca^2+^ levels, reactive oxygen species (ROS), and endoplasmic reticulum (ER) stress to induce the NP CS [20].

Similarly, compressive stress can induce NP CS [21,22,23,24]. Prolonged exposure of cyclic mechanical tension led to DNA damage and induction of premature senescence of NP cells through the activation of the p53-p21-Rb pathway independent of oxidative stress [25]. The expression of membrane receptor G protein-coupled receptor 35 (GPR35) was higher in NP tissues from degenerated discs [26]. In NP cells, mechanical compression induced the intracellular calcium levels via GRP35 to upregulate ROS production, leading to disc cell oxidative damage [26]. Ke et al. reported that compression stress in human NP cells induces the Rho/ROCK1/p-MLC pathway, which induces myosin IIA interaction with actin and reduces the interaction of myosin IIB and actin. This actomyosin cytoskeleton remodeling was involved in regulation of NP CS [22].

Mechanical stress via compression is also associated with disc CS through altered mitochondria function and autophagy. A mechanistic study in rat tail NP cells revealed that the increased mitochondrial injury and oxidative stress-associated senescence occurred after compression overload, and this mitochondrial injury/oxidative stress is regulated by macrophage migration inhibitory factor (MIF) mediated mitophagy in NP cells [21]. Wang et al. showed that in compression induced NP cells, premature senescence is mediated via Sirtuin 1 (SIRT1) regulated PTEN-induced kinase 1 (PINK1)-dependent mitophagy where SIRT1 plays a protective role [23]. Huang et al. reported that compression induces senescence signaling in rat NP cells via PINK1/Parkin-mediated activation of mitophagy [27]. Similarly, high mechanical tension promoted AF cell senescence through inhibition of autophagy [28].

**Figure 2:**
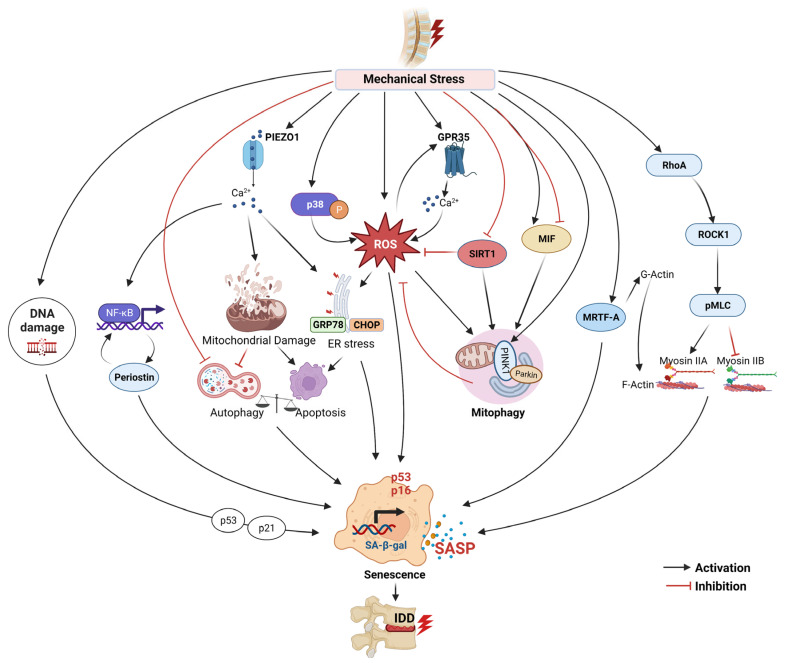
Proposed mechanisms of cellular senescence activation in the intervertebral disc by mechanical stress from reported findings. Chronic abnormal mechanical stress is a major contributor of age-associated for IDD. Mechanical stress including compression or tensile forces can induce DNA damage leading to activation of the p53-p21^CIP1^ senescence pathway. Mechanical stress also increases expression of Piezo1 ion channel resulting in an increase in intracellular calcium level which leads to NF-κB activation, mitochondrial damage, and endoplasmic reticulum (ER) stress. NF-κB activation mediates secretion of periostin by NP cells and promotes cellular senescence (CS) and catabolic processes, creating a NF-κB-periostin activation loop to further amplify the senescence process. Mitochondrial damage on the other hand, inhibits autophagy and induces apoptosis which leads to disc CS. The ER stress activates GRP78 and CHOP, which induce apoptosis and senescence of disc cells. Mechanical stress also induces ROS production regulating the disc cell senescence directly or through ER stress. It is reported that mechanical stress activates ROS production through p38 MAPK or G protein-coupled receptor 35 (GPR35) mediated rise in intracellular calcium level or via sirtuin1 (SIRT1) inhibition. The SIRT1 expression is reduced during mechanical stress thereby inhibiting mitophagy. Mitophagy is activated via macrophage migration inhibitory factor (MIF) or direct activation of PTEN-induced kinase 1 (PINK1). The MIF1 is upregulated by moderate compression stress but downregulated with chronic mechanical compression. Inhibition of autophagy or mitophagy upregulates disc CS. Additionally, actin cytoskeleton reorganization is involved in disc CS. Compression stress activates the RhoA-ROCK1-pMLC pathway to inhibit F-actin interaction with myosin IIA and induce its interaction with myosin IIB leading to an imbalance, which activates senescence. The actomyosin cytoskeleton remodeling is dependent on nuclear translocation of myocardin-related transcription factor A (MRTF-A) in the disc cells. (Figure created with BioRender.com, accessed on 8 March 2023).

Further, high-magnitude compression caused SIPS of NP cells through the p38-ROS signaling pathway [29]. Additionally, simulated microgravity can induce senescence of disc cells [30]. The IVD cells exposed to simulated microgravity using a random positioning machine increased the population of SA-β-gal positive cells, although the exact mechanism is unknown [30]. Taken together, these reports suggest that different forms of mechanical stress can activate biological signaling events leading to CS. Based on these reported findings, we summarized the possible intracellular regulatory mechanisms of CS in response to mechanical stress (Figure 2).

### 4.2. Genotoxic Stress

**Figure 3:**
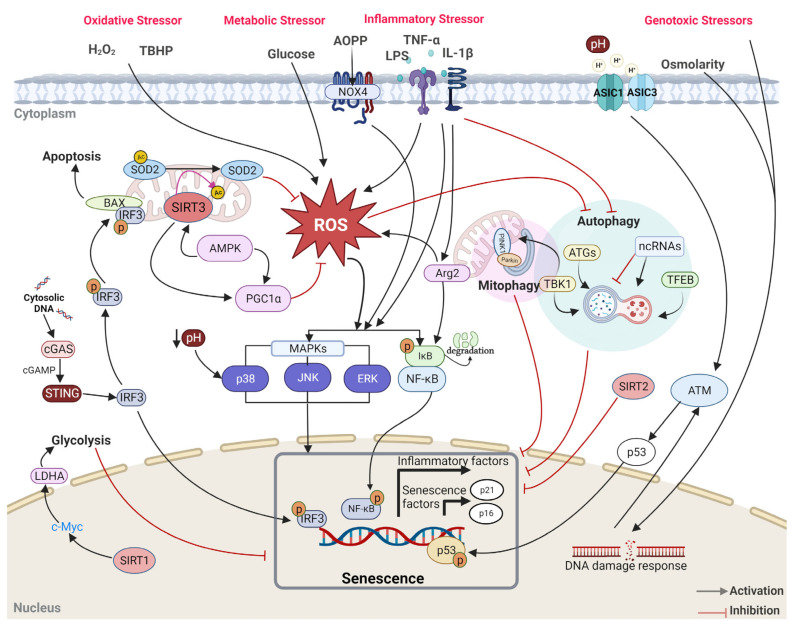
Proposed signaling pathways during cellular senescence activation in intervertebral discs based on reported findings. Oxidative stress is one of the major factors inducing cellular senescence (CS) in intervertebral disc. Accumulation of reactive oxygen species (ROS) occurs from oxidative stress, metabolic stress such as hyperglycemia, or inflammatory stress signaling mediated through toll like receptors (TLRs) or cytokine receptors. An increase in ROS activates MAPK signaling or NF-kB signaling to induce CS and senescence associated secretory phenotypes (SASPs). Secretion of SASP factors further amplify the senescence process through cytokine receptor mediated signaling. Arginase II increases ROS production and activates NF-kB signaling. The AOPPs activate the MAPK and NF-kB signaling pathways through NOX4 to drive disc CS. The levels of sirtuins are reduced in IDD whereas activation of sirtuins is beneficial against disc degenerative changes. The SIRT1 modulates senescence through c-Myc-LDHA mediated glycolysis. The SIRT3 modulates senescence through the AMPK-PGC1α pathway or deacetylation of SOD2. Furthermore, increase in ROS modulates the autophagic pathway thereby affecting CS. Autophagy is downregulated in IDD and several autophagy related genes (ATGs) and other signaling molecules such as noncoding RNAs (ncRNAs), transcription factor EB (TFEB), TANK binding kinase 1 (TBK1) mediated autophagy or mitophagy activation, can reduce senescence of disc cells. Genotoxic and metabolic stressors induce DNA-damage response mediated CS activation via the ATM-p53 pathway. Additionally, cytosolic DNA is sensed by cGAS leading to activation of the cGAS-STING pathway in which activation and nuclear translocation of IRF3 contributes to senescence of the disc cells. In addition, activated IRF3 induces the Bax-mediated apoptotic pathway. Change in pH activates the p38 MAPK mediated senescence pathway or is sensed by ASIC1 or ASIC3, which then directly activates the senescence pathway. For clarity, not all signaling pathways relating to disc CS are included in this figure. Please refer to text for details. AOPPs; Advanced oxidation protein products, ASIC; Acid-sensing ion channels, AMPK; AMP-activated protein kinase, IDD; intervertebral disc degeneration, PGC1α; Peroxisome proliferator-activated receptor-γ coactivator 1 alpha, IRF3; Interferon regulatory factor 3. (Figure created with BioRender.com, accessed on 8 March 2023).

Persistent DNA-damaging stress perturbs genomic stability, which activates cellular failsafe programs like apoptosis or senescence to prevent propagation of cells with damaged genomes. The first direct demonstration of DNA damage-induced disc CS was reported in the *Ercc1^−/Δ^* mouse model of accelerated aging due to DNA repair deficiency. *Ercc1^−/Δ^* mice exhibit even greater CS and premature proteoglycan loss in their IVDs with exposure to genotoxic stressors (ionizing radiation (IR) and chemotherapeutic agents) [31,32]. Two chemotherapy drugs used to mitigate the spread of damaged genomes—both Cisplatin [31] and Bleomycin [33] induce CS but do so through different pathways. Recently, the authors reported that exposure to IR induced the expression of p21^CIP1^ leading to CS with increased matrix catabolism in rat AF tissue [34]. In human NP cells, exposure to IR induced the CS which was comparable with the CS induced by overexpression of p16^INK4a^ or consecutive replications, indicating NP cells express a similar transcriptional profile of genes associated with IVD degeneration, irrespective of the senescence-inducing stress [14]. Moreover, in bovine NP cells cultured under both classic (normal osmolality, hyperoxia, high glucose concentration and in the presence of serum) and IVD (hyperosmolality, low oxygen and glucose concentration and in the absence of serum) conditions, IR exposure and consecutive subcultures led to the CS [35]. These studies suggest that genotoxic stress is a risk factor for developing IDD, especially among the patients undergoing cancer treatment. Signaling pathways involved in CS activation are shown in Figure 3.

### 4.3. Oxidative Stress

Aged and degenerated IVDs have increased oxidative stress and damage, possibly due to the decreased ability to repair damage [36]. The ROS contributes to DNA damage to induce CS, and molecular signaling modulation related to ROS can be targeted to control IVD senescence and degeneration [37,38]. Nucleus pulposus cell senescence is associated with increased ROS in IDD [39]. The ROS are generated in IVD through signaling pathways (i.e., inflammatory or mechanical stress). Mechanical stress from ECM stiffness can lead to increased ROS level and lead to CS. Mechanistically, ROS and increased intracellular Ca^2+^ levels activate ER stress, which is responsible for driving senescence and apoptosis [20]. The level of arginase II, an enzyme which catalyzes the hydrolysis of L-arginine to L-ornithine and urea, is increased in IDD tissue as compared to normal tissue and is positively related to increased ROS levels [40]. Increased levels of ROS and activated NF-κB have been shown to induce CS [40].

The cyclin-dependent kinase inhibitor p16^INK4a^ is involved in the regulation of NP CS through increased ROS [41]. The SIRT3 protects against premature CS induced by ROS via AMP-activated protein kinase (AMPK)/peroxisome proliferator-activated receptor-gamma coactivator (PGC)-1α [42]. Furthermore, oxidative stress is reported to induce CS by inhibiting circular RNA (circKIF18A) [39]. Mechanistically, circKIF18A suppressed the proteasomal degradation of mini-chromosome maintenance complex component 7 (MCM7) [39]. In addition, other exogenous oxidative stressors such as tert-butyl hydroperoxide (TBHP) are widely used in NP cells to induce premature CS signaling in cultured cells [43,44,45]. The DNA damage caused by oxidative stress is sensed by the stimulator of interferon genes (STING) pathway to regulate senescence in NP cells [46]. Expression of STING was upregulated in human and rat degenerated NP tissue, and promoted senescence, apoptosis, and ECM degradation [46]. In addition, serial passage of NP cells grown on monolayer culture induced oxidative stress and senescence [47], suggesting the crosstalk between replicative senescence and oxidative stress.

Advanced oxidation protein products (AOPPs) are novel biomarkers of oxidation-mediated protein damage and are involved in various pathological changes, including degenerative diseases such as osteoarthritis [48]. The AOPPs are increased in rat lumbar and caudal degenerative discs, and exposure of AOPPs upregulate senescence markers (p53, p21 and p16^INK4a^) and pro-inflammatory cytokines (IL-1β, TNF-α) in a rat model of IDD using AF cells [49]. Mechanistically, AOPPs induce senescence via NADPH oxidase and NOX4- mitogen-activated protein kinases (MAPK) activation in AF cells [49]. These studies suggest that oxidative stress from exogenous or endogenous sources can induce CS through several molecular signaling pathways.

### 4.4. Inflammatory Stress

Intervertebral discs degeneration is associated with increased inflammation [50]. There is a significant increase in inflammatory markers in degenerative IVDs, including the pro-inflammatory cytokines TNF-α, IL-1β, and IL-6. Inflammation promotes disc ECM catabolism leading to structural change in the disc. Although the role of inflammatory stress in contributing to disc degeneration is well documented, the role of inflammatory stress in disc CS is unclear. Stimulating NP cells with inflammatory cytokines such as IL-1β or TNF-α can induce premature or acute senescence as marked by increased level of SA-β-gal and other senescence markers p16^INK4a^, p21 and p53, and loss of proliferation [51,52,53,54]. In addition, senescent cells induced by TNF-α treatment activate the senescence of healthy cells through phosphorylation of signal transducer and activator of transcription 3 (STAT3) and the paracrine effect via IL-6 secretion [55].

The ECM derived matrikines induce inflammation in the tissue microenvironment where one of the key matrikine N-acetylated proline-glycine-proline (N-Ac-PGP) is derived from collagen via MMP8, MMP9 and prolyl endopeptidase (PE) cleavage. The level of N-Ac-PGP is positively correlated with the degree of IDD [56] and is involved in the regulation of CS. In replicative senescent rat NP cells, N-Ac-PGP enhanced DNA damage to activate the p53-p21 pathway and promoted ROS production to upregulate p16^INK4a^ expression, via the CXCR1 chemokine receptor [56]. In vivo, transplant of N-Ac-PGP-treated NP cells to rat disc resulted in degenerative changes in the tissue that led to IVD collapse [56].

Inflammatory signaling activated by toll-like receptors (TLRs) also contributes to IDD [57]. Although several TLRs are detected in isolated human IVD cells, expression of TLR-1/2/3/4 and 6 was found to be dependent on degree of IDD [58]. Activation of human IVD cells with TLR-2/6 agonists for up to 48 h can induce senescence [59]. In addition, stimulation with the TLR-4 ligand lipopolysaccharide (LPS) for 24 h increases the expression of p16^INK4a^ and p21 to induce senescence in human NP cells from IDD patients [60]. In AF stem cells, inflammatory signaling activated by LPS for 5 days induced the release of HMGB1 from the nuclei and appears to drive CS [61]. Taken together, these studies suggest that inflammatory signaling activated by a wide variety of stimuli such as DAMPs or PAMPs can induce senescence in disc cells. Although most in vitro studies showed an acute increase in CS markers by inflammatory stimuli, the roles of specific receptor mediated signaling pathways in driving CS are still poorly understood.

### 4.5. Metabolic Stress

Metabolic alteration in aged cells leads to accumulation of metabolites and harmful byproducts resulting in the physiological changes such as increased acidosis, hyperglycemia, and hypoxia to trigger CS [62,63]. The IVD microenvironment is predominantly avascular and is characterized by high acidity, low glucose, hypoxia, and hyperosmolarity. IVD is under constant nutritional restriction with its ECM homeostasis maintained by a low number of cells [64]. The normal pH in healthy disc is 7.1, which drops to 6.5 or less in the degenerated discs due to accumulation of lactic acid and failure of proton (H^+^) processing and transport [65,66]. Acid-sensing ion channels (ASICs) are activated in response to change in pH with levels of ASIC1 and ASIC3 increasing in degenerated human NP cells [67]. Nucleus pulposus cells cultured in acidic condition (pH 6.6) for two days triggered the upregulation of ASIC1 and ASIC3 leading to cell cycle arrest and senescence (increased SA-β-gal, Rb1, p53, p21 and p16^INK4a^) and SASP (increased IL-6, IL-8), which were rescued by treatment with ASIC inhibitors [67]. In rat NP cells, the acidic environment promoted CS through activation of the p38 MAPK pathway [68]. Hyperosmolarity was reported to induce DNA double strand breakdown and cell cycle arrest in NP cells through activation of the ATM-p53-p21^WAF1^ axis leading to hypophosphorylation of Rb [69]. The combination of low glucose, hypoxia, high osmolality, and absence of serum was anti-proliferative for young NP cells with senescence phenotype [35]. Therefore, maintenance of disc microenvironment likely is essential to control disc CS and age-related IDD.

Hyperglycemia or high glucose level accelerated senescence and degeneration in disc cell cultures [70,71] and in diabetic rats [72]. Diabetes mellitus is one of the predisposing factors for IDD in human as the patients with disc disease have a statistically significant increased incidence of diabetes mellitus [73]. Cell culture studies demonstrated that hyperglycemia causes the premature senescence of NP cells through increase in ROS level and activation of the NF-κB pathway [71,72,74,75]. These data suggest that diabetic patients are at higher risk of CS and age-related IDD. With aging, the risk of metabolic diseases increases, which possesses a threat to disc health.

## 5. Signaling Molecules/Pathways

### 5.1. p53-p21^CIP1^-Rb and p16^INK4a^-Rb Pathways

The p53-p21^CIP1^-Rb and p16^INK4a^ -Rb tumor suppressor pathways can operate separately or cooperatively to block cell cycle progression and induce senescence [76]. Activation or suppression of these pathways are dictated by stress signal, type of tissue, or species of origin [77]. It has been reported that the p53-p2^CIP1^ pathway plays a major role in initiation of senescence while the p16^INK4a^-Rb pathway is required for stable cell cycle arrest and senescence [78]. The p53 is activated via post translational modifications such as phosphorylation, methylation, acetylation, sumolyation, etc. [78,79]. The major upstream effector of p53 includes the ATM-Chk2 or ATM-Chk1 pathway, which then activates p53 to induce downstream effectors (i.e., p21^CIP1^, an endogenous cyclin-dependent kinase inhibitor (CDKi) that plays a key role in p53-mediated cell cycle arrest) [80]. Alternatively, senescence stress-induced activation of the CDKi p16^INK4a^ leads to inactivation of CDK4/6, which lead to hypophosphorylation of Rb which inhibit the regulation of E2F and other transcriptional factors to induce cell cycle arrest and senescence. Crosstalk between the p53 and p16^INK4a^ pathways influences the outcome of cell cycle arrest and senescence. Currently there are no markers specific for senescent cell-cycle arrest as p16^INK4a^ is also expressed in certain non-senescent cell types and absent in some types of SnCs [6,76]. Indeed, it appears that there are distinct subsets of p16^INK4a^ and p21^Cip1^ SnCs [81,82]. Other than promoting cell cycle arrest, p53 also mediates the SASP response in SnCs. Typically activated by DNA damage which triggers DAMPs or type 1 interferon responses, SASP is controlled by activation of several signaling molecules such as NF-κB, GATA binding protein 4 (GATA4), mammalian target of rapamycin (mTOR), and p38 MAPK [83,84,85].

The p53 and p16^INK4a^ pathways have been shown to activate senescence in NP cells [31]. The level of p16^INK4a^ expression has also been shown to increase with aging in human disc and animal models of aging [8,13,86]. Inflammation induced by TNF-α or IL-1β increases the p16^INK4a^, p21^CIP1^, and p53 expression in NP cells [51,52,53,54,55]. In cultured NP cells, a combination of low glucose, hypoxia, high osmolality, and absence of serum induces the expression of p16^INK4a^, p21^CIP1^, and intercellular adhesion molecule-1 (ICAM-1) [35]. These studies suggest that the p52-p21^CIP1^ or p16^INK4a^ pathways are activated in disc cells by various stresses to induce premature or acute senescence. However, whether these cells really became senescent remained unclear as few reported studies performed vigorous testing for CS with multiple confirmatory senescence and SASP biomarkers. Moreover, it is still not clear if there are distinct p21^CIP1^-driven and p16 ^INK4a^-driven senescent cell populations in disc tissue.

### 5.2. MAPK and NF-κB Signaling

The MAPK pathway is involved in the signal transduction of cellular growth, development, and differentiation when cells respond to extracellular signals such as hormones, growth factors, cytokines, and environmental stress [87]. Major MAPK cascades identified in eukaryotic cells are extracellular signal-regulated kinase (ERK), c-Jun NH2-terminal kinase (JNK), and p38 MAPK [88]. Multiple studies have implicated MAPK signaling in IDD. Activation of ERK, JNK or p38 is involved in the stress-induced premature CS of NP or AF cells [2]. The activation of heat shock protein 70 (HSP70) downregulated the JNK/c-Jun pathway in TBHP-stimulated NP stem cells to protect against apoptosis and senescence [89] suggesting a role for JNK in senescence. In addition, substrate stiffness induced NP cell senescence via the p38 MAPK pathway in an animal cell model that can be inhibited by lysyl oxidase (LOX) via regulating the integrin β1-p38 MAPK pathway [90]. The precise mechanisms behind how activation of MAPK signaling regulate senescence of disc cells is not clear, and future research using proper animal models as well as inhibitors are needed to confirm whether MAPK could be a direct target to reduce disc cell senescence and IDD.

The transcription factor NF-κB is central to the cellular response to damage, stress, and inflammation through regulating expression of a wide variety of genes related to immunity, inflammation, and cell cycle [91]. In response to diverse stress stimuli, the canonical NF-κB signaling pathway is activated by proteasomal degradation of IκBα triggered by the IκB kinase (IKK) complex. This results in the nuclear translocation of NF-κB to transcriptionally activate genes for subsequent regulation of inflammatory responses [91]. The NF-κB is activated in IVDs in naturally aging mice and in the *Ercc1^−/Δ^* mouse model of accelerated aging [92]. The level of phospho-p65 is higher in NP cells from degenerated disc as compared to normal NP cells [93,94,95]. In NP cells, external stimuli such IL-1β or cisplatin induce CS via NF-κB activation [31,52]. The NF-κB is recently reported to promote p16^INK4a^ expression in human NP cells by activating the *CDKN2A* [41], suggesting a regulatory role of NF-κB in disc CS.

### 5.3. Sirtuins

Sirtuins (SIRTs) are a family of nicotinamide adenine dinucleotide (NAD+)-dependent deacetylases responsible for sensing NAD+ fluctuations and regulation of energy and metabolic homeostasis as well as redox balance [96]. Sirtuins are closely related to many cellular functions involved in longevity, immune and inflammatory responses, DNA damage repair, etc. [97]. Of the seven known SIRTs [97], only a few have been studied in the context of CS and IDD.

Among the best studied member of sirtuin family is SIRT1 [98]. As a metabolic/nutrient sensor, SIRT1 is localized in the nucleus but can shuttle between the nucleus and cytoplasm under specific conditions [99]. It is highly expressed in healthy human discs but its level decreases in degenerated discs [100]. In degenerated discs, reduced SIRT1 expression leads to inhibition of c-Myc mediated lactate dehydrogenase A (LDHA) expression, resulting in reduced glycolysis pathway and ultimately increased CS [100]. Oxidative stress increases phosphorylation of forkhead box protein O1 (FOXO1), decreasing its nuclear translocation and inhibits the SIRT1 pathway via activating the Akt [101]. Activation of SIRT1 suppresses CS of NP cells [101,102].

Sirtuin 2 is mainly localized in the cytosol but can translocate into nucleus. It regulates several physiological functions, including but not limited to mammalian metabolism, cell differentiation, mitophagy, and cardiac homeostasis [103,104,105]. In the context of IDD, the level of SIRT2 is decreased in degenerated discs and senescent NP cells [106]. Senescent NP cells have increased p53-p21^CIP1^ and ROS levels, and reduced levels of the antioxidants SOD1/SOD2—suggesting a potential role of SIRT2 in modulating disc CS and IDD development and a possible therapeutic target [106].

Sirtuin 3 is a major mitochondrial deacetylase that regulates mitochondrial function through NAD+ dependent protein deacetylation. It plays a key role in metabolic, cardiovascular, and neurodegenerative diseases [107]. As with SIRT1 and SIRT2, the level of SIRT3 is decreased in degenerated NP tissues (both human and rat) [42,45]. The SIRT3 expression is increased in oxidative stress- (H_2_O_2_ or TBHP) induced senescent NP cells [42,45]. However, overexpression or activation of SIRT3 protected rat NP cells from developing oxidative stress-induced senescence [42]. Part of the protective mechanism is mediated through SIRT3 deacetylation of SOD2 to reduce ROS accumulation [42]. Furthermore, the AMPK/PGC-1α signaling pathway is involved in the regulation of SIRT3 in H_2_O_2_- [42] and TBHP-induced [45] premature senescence of NP cells. This suggests a significant role of the AMPK/PGC-1α signaling pathway in regulation of SIRT3 in CS and IDD.

Sirtuin 6 is known to regulate lifespan in mice as SIRT6 overexpressing transgenic mice live longer than wild-type littermates [108]. It regulates senescence in several biological processes [109,110,111]. The level of SIRT6 is reduced in senescent NP cells both in vivo and in vitro [54]. Mechanistically, SIRT6 overexpression attenuated the senescence including replicative senescence and inflammatory stress-induced (IL-1β stimulated) premature senescence as marked by the level of p53, p16^INK4a^, and p21^CIP1^ as well as SA-β-gal staining [54]. Autophagy inhibition abolished the anti-senescence activity of SIRT6, which suggests autophagy plays a vital role in SIRT6-mediated regulation of senescence and matrix degrading enzymes (MMP13, MMP13, ADAMT4, and ADAMT5) in NP cells [54]. Finally, these in vitro findings were supported by in vivo results demonstrating SIRT6 overexpression mitigates IDD induced by disc puncture in rat [54]. These studies suggest that SIRT6 is a potential therapeutic target to attenuate disc CS and degeneration.

### 5.4. Wnt/β Catenin Signaling

Wnt/β-catenin signaling regulates cell proliferation, metabolism, growth, and development [112]. Although dysfunction of Wnt/β-catenin signaling has been observed in various degenerative diseases including IDD in the context of apoptosis and other cellular processes [113,114,115,116], the role of Wnt/β-catenin in disc senescence and IDD is not well understood. Tandem mass spectrometry analysis and other molecular techniques demonstrated that the Wnt/β-catenin pathway proteins are upregulated in degenerative NP cells and tissues [117,118]. In IL-1β treated NP cells, the senescence and degenerative changes are driven by Rac1 through activation of the Wnt/β-catenin pathway while use of a Rac1 inhibitor (NSC23766) could alleviate the degenerative changes associated with IL-1β-treated NP cells as well as in a puncture-induced IDD rat model [117]. Mechanical compression of rat NP cells for up to 48 h induced Wnt/β-catenin signaling and enhanced autophagy in NP cells, resulting in reduction in apoptosis and senescence [119]. The activation of Wnt/β-catenin increased cell survival whereas its excessive activation led to increased cell death [119]. These studies implicate Wnt/β-catenin as a potential important regulator of disc CS and degeneration.

### 5.5. PI3K/Akt/mTOR Signaling

Regulated via a multistep process, the highly conserved phosphatidylinositol 3-kinase (PI3K)/Akt signaling pathway plays a significant role in CS and cellular activities, as well as diseases relating to IDD, cancer, and aging [120,121,122]. The PI3K/Akt signaling pathway is activated by TNF-α in NP cells, and treatment with the PI3K inhibitor LY294002 showed protective effects against TNF-α-induced premature senescence, suggesting its role in mediating activation of CS and disc degeneration [123]. High glucose-induced ROS has been reported to promote premature senescence of NP cells via the PI3K/Akt pathway [75]. Intriguingly, activation of the PI3K/Akt pathway alleviated replicative senescence of human NP cells [124]. In addition, bone morphogenetic protein-7 (BMP-7) downregulated the expression of p16^INK4a^ and p53 through PI3K/Akt signaling, given that LY294002 partially blocked the preventive effects of BMP-7 [124]. These reports suggest that PI3K/Akt play a role in regulating disc CS and IDD. Future studies are warranted to further confirm PI3K/Akt as a senotherapeutic target for age-related IDD.

The mTOR is a serine/threonine protein kinase regulating cell growth and proliferation and is involved in the aging process. It exists in two structurally and functionally distinct complexes: (1) mTORC1 comprising the regulatory-associated protein of mTOR (RAPTOR) and (2) mTORC2 containing the rapamycin-insensitive companion of mTOR (RICTOR). The mTOR signaling is detectable in human disc tissues [125,126], and is known to regulates disc health through the regulation of autophagy [127]. The mTOR signaling has been demonstrated to regulate disc CS and IDD [41,125,126,127] implicating mTOR as a therapeutic target for IDD, as discussed in senotherapy section below.

### 5.6. Non-Coding RNAs and Epigenetic Mechanisms

Aging-associated diseases are accompanied by altered epigenetic gene regulation by non-coding RNAs, DNA methylation, chromatin remodeling, and histone modification. Non-coding RNAs (ncRNAs) are functional RNA molecules that are not translated into proteins, examples of which include micro RNAs (miRNAs), ribosomal RNAs (rRNAs), circular RNAs (circRNAs), long noncoding RNAs (lncRNAs), and transfer RNAs (tRNAs). The ncRNAs can regulate or influence CS alone or in concert with each other. Several miRNAs have been identified to modulate expression of the key senescence biomarkers p53-p21 or p16^INK4a^ [128,129]. Direct and indirect regulation of senescence pathways by miRNAs have also been reported [128]. Several miRNAs are upregulated [130,131] or downregulated [60,132,133,134,135] during IDD. In addition, circRNAs and LncRNAs, at times acting in concert with miRNAs regulate NP cell function and IDD [118,136].

The non-coding RNA, CircERCC2 was found to be downregulated in degenerated disc tissue, as opposed to miR-182-5p that was upregulated. Both of these ncRNAs were reported to regulate mitophagy, apoptosis, and senescence in NP cells [131]. Mechanistically, miR-182-5p targeted SIRT1 by binding to SIRT1 mRNA, and circERCC2 targeted miR-182-5p/SIRT1 cascade to control the NP cell functions [131]. In addition, expression of lncRNA H19 and Wnt/β-catenin signaling were upregulated in IDD tissue [118]. In H_2_O_2_-induced senescence of NP cells, miR-22 acted as a negative modulator of H19 [118]. Here lncRNA H19 has been hypothesized to compete with lymphoid enhancing factor-1 (LEF1) for miR-22 to regulate downstream Wnt/β-catenin signaling [118]. Reduced in IDD, miR-4769-5p targets the transmembrane serine protease (Hepsin) to inhibit its expression and thereby inhibits NP CS [133]. Elevated in expression in degenerated discs, lncRNA TRPC7-AS1 acted as a competing endogenous RNA (ceRNA) and reversed the effect of miR-4769-5p on Hepsin to modulate NP CS and functions [133]. Several ncRNAs appear to have a regulatory role in CS via the regulation of autophagy [43,137,138], which will be discussed in detail in a separate section below. These studies suggest that certain non-coding RNAs act in tandem or in concert with each other to modulate cellular processes linked to CS during disc degeneration. Table 1 summarizes studies on ncRNAs in relation to disc CS and IDD.

Both DNA methylation and histone modifications control the chromatin architecture to regulate the gene expression programming, and these epigenetic mechanisms are closely connected to senescence [140,141]. For example, ALKBH5-, a core component of demethylase mediated mRNA demethylation (i.e., *N6*-methyladenosine (m^6^A) modification) plays a key role in NP CS and IDD [13]. The ALKBH5 was increased in TNF-α-induced premature senescent NP cells and in degenerated NP tissue [13]. Mechanistically, m^6^A hypomethylation of DNMT3B enhanced the stability of DNMT3B mRNA. Increased iDNMT3B expression reduced the expression of E4F1 level upon DNA methylation to cause cellular arrest and senescence of degenerated NP cells [13]. Furthermore, the m^6^A modification of lncRNA NORAD played a major role in activation of senescence in NP cells [139]. In SnCs, lysine demethylase 5a (KDM5a) levels are reduced thereby enhancing the H3K4me3 modification of WTAP, which is a core of methyltransferase complex, to induce its expression. Analysis of TNF-α-treated NP cells showed an increase in the level of WTAP mediated m^6^A modification of lncRNA NORAD and an increase in binding of PUM1/2 to E2F3 mRNAs [139]. This binding caused degradation of the E2F3 transcript and blocked the cell cycle to promote senescence of NP cells [139]. These reports suggest the crosstalk of methylation regulation by histone modification, m6A and DNA methylation during establishment of disc CS.

### 5.7. Autophagy/Mitophagy

Autophagy is an intracellular catabolic process that eliminates unnecessary or dysfunctional components through lysosomal degradation and recycling of cytoplasmic cargos to maintain the intracellular quality control and homeostasis [142]. A prominent type of autophagy, macroautophagy is a multistep process characterized by initiation of double membrane phagophore, closure to form an autophagosome, and fusion with lysosome to become an autolysosome for the degradation of cytoplasmic cargo. Autophagy has been implicated as a pro-longevity mechanism [142] and in IDD [143,144]. The level of autophagy in the human NP and AF tissue is reduced with aging [145].

Autophagy related genes (ATGs) are evolutionarily conserved and central to the autophagy process. The ATG7, one of the core ATG proteins, is downregulated in NP cells treated with high glucose [146]. High glucose treatment for a short time (6 h) reduced the human antigen R (HuR) expression leading to inhibition of ATG7 and autophagy to induce CS [146]. Overexpression of ATG7 ameliorated diabetic-IDD in rats, also reducing CS as indicated by reduction in the expression of p16^INK4a^ in disc tissue [146]. In another study, ATG5 was activated under nutritional deprivation or inflammatory stress [145]. Treatment with IL-1β or serum-free culture conditions induced the expression of ATG5 and autophagy in human NP cells while siRNA-mediated knockdown of ATG5 caused inhibition of autophagy to induce CS by increasing the p16^INK4a^, p21, and p53 expression. However, matrix catabolism and the MAPK-signaling pathways were unaffected by ATG5 knockdown in these reported studies [145,147].

Autophagy in IVD is regulated by several ncRNAs. For example, in NP cells treated with TBHP to induce oxidative stress, inhibition of miR-130b-3p increased autophagy via ATG14 and AMPK, leading to suppression of cell apoptosis and senescence, reduction in the level of MMP13 and ADAMTS4, and induction of collagen II and aggrecan levels [43]. miR-217-induced the autophagy in TNF-α-stimulated NP cells through the enhancer of zeste homolog 2 (EZH2)/FOXO3 signaling [137]. The TNF-α treatment of NP cells reduced miR-217 expression. In addition, miR-217 delivered to NP cells via mesenchymal stem cell extracellular vesicles (MSC-EVs) bind to EZH2 to inhibit the histone methylation of EZH2 on the FOXO3 promoter to activate the autophagy and inhibit apoptosis and CS [137]. The lncRNA HOTAIR regulates senescence by modulating autophagy in NP cells [138]. The HOTAIR mRNA expression increased and was positively correlated with IDD grades. Intriguingly, there appears to be activation of both autophagy and senescence by HOTAIR overexpression, and inhibition of autophagy attenuated HOTAIR-induced senescence in NP cells. This suggests that activated autophagy promotes NP CS under this condition [138]. Importantly, HOTAIR silencing with siRNA inhibited IDD in rats [138].

The transcription factor EB (TFEB), identified as a master regulator of autophagic flux, plays a protective role in mitigating IDD [44,148]. The TFEB is downregulated in degenerated rat NP cells and in TBHP-exposed NP cells [148]. The TFEB overexpression ameliorated IDD and prevented TBHP-induced increased expression of SA-β-gal, p16^INK4a^, IL-6, and MMP-13 in rat NP cells. Moreover, TFEB-overexpression rescued the blockage of autophagic flux and lysosomal dysfunction in rat NP cells [148]. The TFEB was reduced in TBHP-treated NP cells, causing the blockage of autophagic flux, which lead to induction of apoptosis, senescence, and ECM degradation [44]. The TFEB activation by apigenin reversed the TBHP-induced p21 and p16^INK4a^ expression and SA-β-gal activity, suggesting the role of TFEB in CS and IDD [44]. Autophagy can also be activated by physical exercises, thereby alleviating CS and apoptosis [149]. Physical exercise was recently reported to inhibit the development of age-related IDD in mice and rats through induction of FNDC5/irisin mediated autophagy activation [149]. Mechanistically, FNDC5/irisin activated autophagy through the AMPK/mTOR signaling pathways to inhibit CS (e.g., increased expression of p16^INK4a^ and SA-β-gal positive cells) and apoptosis (e.g., increased cleaved-caspase3 and TUNEL-positive cells) in NP cells and ameliorated senescence and IDD in a rat model [149]. These studies reveal the connection between autophagy and CS through a complex network of gene products and pathways in disc cells exposed to different stressors.

Mitophagy, a selective autophagic process of clearing dysfunctional mitochondria, plays a critical role in cellular responses against various stressors such as oxidative stress. Dysfunctional mitochondria formed under stressful conditions are recognized by PINK1 and Parkin to induce mitophagy. Under mitochondrial depolarization, PINK1 accumulates and is activated on the outer mitochondrial membrane leading to recruitment and activation of Parkin, the E3 ubiquitin ligase. Activated Parkin induces the ubiquitination of outer mitochondrial membrane proteins that can be recognized by several autophagic receptors including SQSTM1/p62, NDP52, and optineurin [150]. The expression of PINK1/Parkin is increased in degenerative NP tissues [151] and in NP cells under compression stress or treated with TNF-α or H_2_O_2_ [21,27,152], suggesting that mitophagy is activated in stressed disc tissue and cells. Induction of premature senescence of NP cells during compression stress has been reported to occur via activation of the PINK1/Parkin pathway-mediated activation of mitophagy [27]. The TANK-binding kinase 1 (TBK1) belonging to the IKK-kinase family of kinases is involved in innate immune signaling pathways and plays a major role in autophagy and mitophagy [153]. The level of TBK1 is reduced in IDD and in senescent NP cells from rats and humans [152]. The TBK1 overexpression reduced replicative senescence, and TNF-α-induced senescence and apoptosis [152]. The TBK1 overexpression induced the autophagy-lysosome fusion as seen by LC3-II and LAMP1 immunofluorescence double staining in TNF-α stimulated NP cells. The TBK1 directly phosphorylated p62 at S403 to promote autophagy. The study further showed that phosphorylation of TBK1 specifically induces the Parkin-dependent mitophagy in TNF-α stimulated NP cells. Results showed that overexpression of TBK1 inhibited IDD development in rat models [152]. As mitophagy is involved in the pathogenesis of IDD [154], further investigation is warranted to determine the role of autophagy and mitophagy and their crosstalk with CS during IDD.

## 6. Therapeutics

### 6.1. Senolytics

Senescent cells increase during aging, and elimination of SnCs can prevent or delay aging-associated changes [41,155,156]. In aging mice, systemic clearance of p16^INK4a^positive cells by genetic strategy reduced age-associated IDD [1], suggesting that removal of SnCs can restore tissue homeostasis in aged tissue. Senolytics are a class of therapeutics targeting SnCs to induce apoptosis specifically by inhibiting their pro-survival or anti-apoptotic pathways upregulated during senescence. Several small molecules and drugs have been extensively studied as senolytics in several pathological conditions such as fibrotic pulmonary disease, bone loss, etc. [157,158,159]. However, study of their therapeutic potential in the context of disc degeneration is still in its infancy. 

The kinase inhibitor dasatinib (D) induces apoptosis in SnCs by inhibiting the Src-family tyrosine kinases, while the polyphenol quercetin (Q) does so by inhibiting the anti-apoptotic protein Bcl-xL. The inhibitors D+Q were the first senolytics demonstrated to reduce IDD in the *Ercc1^−/Δ^* mouse model of accelerated aging [160] and to extend healthspan and lifespan in naturally aged mice. Quercetin was reported to prevent IL-1β-induced CS as well as to ameliorate disc degeneration in a puncture-induced rat model through inhibition of the NF-κB pathway and activation of the antioxidant Nrf2 [52]. Intermittent treatment with the D+Q cocktail can restore disc health in naturally aging mice [161]. A transcriptome study revealed that D+Q modulates critical genes associated with aging in NP cells. The D+Q treatment for 6- and 14-months reduced the level of IDD, with decreased senescence markers p16^INK4a^, p19, and SASPs. Treatment with D+Q not only prevented degeneration but also preserved healthy disc ECM and reduced the aggrecan degradation during aging in mice. This study also found that prolonged treatment is well tolerated suggesting the therapeutic potential for D+Q treatment of IDD [161]. These studies also suggest that quercetin alone or combination with dasatinib could be a promising therapeutic agent in the treatment of IDD.

Natural compounds also have been tested as senolytics in IDD models. Morroniside, a major iridoid glycoside of a popular traditional Chinese medicine Fructus Corni, is effective in alleviating IDD features in a H_2_O_2_-induced NP CS cell culture model and in a surgically induced IDD mouse model [162]. Morroniside inhibited the ROS-Hippo-p53 signaling pathway to attenuate CS signaling and prevents matrix degradation to protect against IDD progression [162]. Curcumin is a natural active therapeutic compound used to treat various human disorders, including aging-related pathologies [163,164]. Curcumin exerted anti-apoptotic and anti-senescence effects in TBHP-treated human NP cells and inhibited IDD progression in a rat model [165]. Mechanistically, curcumin restored the TBHP-induced blockage of autophagy flux to inhibit the TBHP-induced apoptosis, senescence, ECM degradation, oxidative stress, and mitochondrial dysfunction in human NP cells [165]. Treatment of human NP cells with curcumin or its metabolite o-vanillin decreased numbers of p16^INK4a^ positive cells and increased numbers of the Ki-67 and caspase-3 positive cells in a monolayer and pellet culture [166]. In addition, curcumin and o-vanillin decreased the inflammatory signaling in NP cells as confirmed by reduced phosphorylation of p65/RelA, Nrf2, JNK and ERK—demonstrating their senolytic and anti-inflammatory properties [166]. A comparative analysis between o-vanillin and the synthetic compound RG-7112 (a potent mouse double-minute two protein inhibitor) revealed that these compounds activate apoptotic pathways to kill senescent IDD cells. In contrast, these compounds were reported to activate proliferation-related pathways in non-senescent disc cells [167]. Both compounds reduced inflammatory cytokines, chemokines, and growth and neutrophilic factors in pellet culture and in the intact human IVDs [167]. In addition, o-vanillin reduced the number of SnCs in cell pellet culture from degenerated discs and inhibited the TLR-2-induced senescence of disc cells isolated from patients with back pain and IVD degeneration [59]. Mechanistically, o-vanillin reduced TLR-2 and p16^INK4a^ co-expressing cells, suggesting that blockage of TLR-2 by o-vanillin reduces the detrimental effect of SnCs [59]. The disc senolytics studies described here are summarized in Table 2.

### 6.2. Other Therapeutic Possibilities

#### 6.2.1. Natural and Synthetic Compounds

Several natural and synthetic agents possessing anti-senescence properties have been reported. Apigenin, a natural flavonoid in multiple plants, vegetables, and fruits, is shown to have preventive effects on IDD by modulating the CS pathway [44]. Apigenin induced the AMPK/mTOR/TFEB signaling pathway to increase autophagy thereby reducing oxidative stress TBHP-induced senescence of NP cells, which mitigated IDD features in both in vitro and in vivo models [44]. Honokiol, a small molecular weight natural compound extracted from the bark of magnolia trees, is reported to activate SIRT3 through the AMPK-PGC-1α-SIRT3 signaling pathway to suppresses premature senescence and apoptosis in TBHP-treated NP cells [45]. Resveratrol, a polyphenol compound found in red wine, is reported to attenuate NP cell apoptosis and senescence induced by high glucose [75]. In oxidative stress-induced premature senescence, resveratrol also inhibited IDD by activating SIRT1 via inhibition of the FOXO1-Akt pathway [101]. The same study revealed that a SIRT1 activator SRT1720 also has an anti-senescence effect in H_2_O_2_-stimulated rat NP cells [101]. In addition, resveratrol inhibited senescence of rat NP cells induced by long term exposure of TNF-α and IL-1β that mimics the chronic inflammatory microenvironment of IDD [175].

Metformin, a well-known hypoglycemic drug, was shown to protect rat NP cells from undergoing senescence upon exposure to oxidative stress by inhibiting expression of the p16^INK4a^, p53, and p21^CIP1^, and cGAS-STING-NF-κB pathways [168]. Metformin inhibited the cGAS-STING pathway through induction of autophagy both in vitro and in vivo [168]. In rat AF cells stimulated with LPS, metformin inhibited the production of PGE2 and HMGB1, as well as CS markers, suggesting its senotherapeutic potential in treating IDD [61,168].

Rapamycin, a specific inhibitor of mTORC1 is a macrolide used clinically to prevent rejection in organ transplantation due to its potent immunosuppressive activity. Interestingly, rapamycin treatment at lower doses decreases certain age-related pathologies and increases lifespan in mice [176]. In AF cells, bleomycin-induced senescence was inhibited by rapamycin treatment [33]. Rapamycin inhibited phosphorylation of S6 induced by bleomycin, which reduced cell senescence, catabolic and inflammatory responses, and stem cell differentiation in AF cells [33]. Rapamycin inhibited the p16^INK4a^ expression in IL-1β treated NP cells and prevented NP disc degeneration by inhibiting ROS levels and mediating cell cycle [41]. Rapamycin also inhibited the IL-1β-induced apoptosis, senescence, and matrix catabolism in human NP cells through autophagy activation [126]. In addition, mTORC1/RAPTOR knockdown decreased the IL-1β-induced SA-β-gal-positive cells and p16^INK4a^ expression in human NP cells through induction of autophagy [126]. Furthermore, the specific mTORC1 inhibitors including rapamycin, temsirolimus and everolimus all protect the human NP cells from IL-1β-induced apoptosis, senescence, and matrix catabolism [125]. However, they also reported that a dual mTOR (mTORC1 and mTORC2) inhibitor INK-128 and a PI3K-dual mTOR blocker NVP-BEZ235 failed to protect against IL-1β-induced apoptosis, senescence, and matrix catabolism [125]. These data suggest that mTOR, specifically mTORC1, is a therapeutic target in CS and IDD.

Growth factors and hormones have been reported to have a senotherapeutic effect in disc cells. Bone morphogenic proteins (BMPs) are multifunctional growth factors that belong to the transforming growth factor-β (TGF-β) superfamily. Osteogenic protein-1 (OP-1), also known as BMP-7, can inhibit subculture- or TNF-α-induced senescence in NP cells [53,124]. In contrast, prolonged treatment of NP cells with a high dose of recombinant human BMP-2 (rhBMP-2) is reported to increase SA-β-gal activity and p53, p21, and p16^INK4a^ expression, [177] suggesting the adverse effects of long-term exposure to growth factors. Omentin-1, one of the adipokines derived from white adipose tissue, attenuated IL-1β-induced CS through activation of SIRT1 [169]. The estrogen receptors ERα and ERβ are expressed in NP cells and it has been reported that in TNF-α-stimulated NP cells, the estrogen hormone 17beta-estradiol (E2) reduced SA-β-gal activity and p53 and p16^INK4a^ expression, inhibited ROS generation and NF-κB activity while increasing telomerase activity [170]. A parathyroid hormone is also reported to block premature senescence by dexamethasone by activating autophagy via mTOR inhibition [171]. As most of these studies have been performed in cell culture, in vivo studies are required to confirm their therapeutic potential.

Several antioxidative and anti-inflammatory agents have been reported to have anti-senescent therapeutic potential in IDD. Polyamines are naturally occurring polycationic alkylamines contributing to oxidative balance [178]. A study found that polyamines levels are reduced—and polyamine oxidase expression is increased—in degenerated NP tissues and rebalancing polyamine metabolism delays IL-1β-induced human NP CS [172]. Oral supplementation of spermidine, a polyamine compound, reduced the senescence and ECM stabilization [172]. Spermidine inhibited the H_2_O_2_ level thereby inhibiting oxidative stress induced DNA damage and NP cell senescence in an age-related IDD mouse model [172]. Similar effects were observed in p16^INK4a^-deficient and aging mice treated with the antioxidant N-acetyl cysteine (NAC) [172]. Oral NAC supplementation reduced disc degeneration and CS in vitro and in vivo [172,174], suggesting that antioxidant supplementation may reduce the effects of aging in IDD. The B-lymphoma Moloney murine leukemia virus integration site 1 (Bmi-1) is involved in cell-cycle regulation and CS by modulating the p16^INK4a^/Rb and p19/p53 pathways. The Bmi-1 protects against oxidative stress and ROS-induced senescence [174]. In vivo studies showed that Bmi-1 deficiency aggravated oxidative stress-induced IDD in mice, which was inhibited by oral NAC supplementation [174]. One study concluded that mitochondrial-derived ROS promotes aging-related IDD [179], but its role in disc CS requires further investigation.

Disc CS can be modulated by several metabolites. Urolithin is a dibenzopyran-6-one derivative produced by intestinal microbial metabolism from foods rich in polyphenols such as ellagitannins and ellagic acid [180]. Urolithin A is reported to inhibit H_2_O_2_ induced premature senescence through the SIRT1/PGC-1α signaling pathway in NP cells as well as in an IDD rat model [173]. Inhibitors of the acid sensing ion channel subunit (ASIC) protein complex reportedly block senescence activating the p53/p21 and p16^INK4a^/Rb1 pathways in NP-MSCs co-cultured under acidic condition [67]. These include the ASIC1 inhibitors psalmotoxin and amiloride used to treat high blood pressure and the ASIC3 channel specific blocker APETx2. Current clinical medicines could have additional indications for reducing CS, which would need to be investigated further.

#### 6.2.2. Genetic Alterations and Other Interventions

Genetic alterations of several signaling molecules are effective in reducing CS and IDD during aging. Bromodomain-containing protein 4 (BRD4), a member of bromodomain and ultra-terminal structure family is expressed in human NP tissues and is positively correlated with IDD severity [181]. The shRNA-mediated downregulation of BRD4 reduced the IL-1β-induced expression of senescence markers p16^INK4a^ and p21 as well as the apoptosis marker caspase-3 in human NP cells [181]. Mechanistically, BRD4 inhibition activated autophagy via the AMPK/mTOR/ULK signaling pathway to reduce senescence and ECM degradation. In a puncture model of IDD in rats, BRD4 inhibition had a protective effect suggesting the therapeutic potential of modulating BRD4 in age-related IDD [181]. Deletion of CDKi p16^INK4a^ in mice reduced aging-associated increase in ROS content in NP cells, reduced DNA damage, and protected against the loss of disc height [41,172]. In the *Ercc1^−/Δ^* mouse model of accelerated aging, heterozygosity of the NF-κB subunit p65 (i.e., *Ercc1^−/Δ^p65^+/−^* mice) reduced age-associated IDD [92]. Similar protective effects were observed with the treatment of the NF-κB activation inhibitor 8K-NBD peptide in *Ercc1^−/Δ^* mice [92]. Silencing caveolin-1, a plasma membrane caveolae reduced the oxidative stress-induced senescence via the regulating p53/p21 signaling pathway in human NP cells [182]. In addition, N-cadherin, an adhesion molecule had a protective effect on cells under high-magnitude compression and high glucose [24,74]. The study showed that overexpression of N-cadherin reduces CS induced by compression [24]. In glucose-induced CS, N-cadherin overexpression reduced the ROS content and the NF-κB pathway signaling to attenuate senescence [74], suggesting that N-cadherin may be a potential therapeutic target but requires further validation.

STING-knockdown using shRNA injection into the disc was shown to alleviate puncture-induced IDD in rats [46]. STING promoted senescence and apoptosis in IVD via the IRF3 pathway, which can be a therapeutic target as suggested by in vivo and in vitro studies [46]. However, a recent study using N153S mice (with constitutively active STING) and STING^−/−^ mice reported that the cGAS-STING pathway is not involved in the regulation of IVD senescence and degeneration [183]. Report showed that constitutive STING activity or the absence of STING in mice has no evidence of accelerated disc senescence or disc degeneration, rather the cGAS-STING pathway was involved in the maintenance of trabecular bone in the vertebrae [183]. It was noted that systemic hypercytokinemia was associated with cGAS-STING activation [183]. These discrepancies between the study reveal the analytical gap between different study tools/methods, which should be identified and acknowledged in future.

Dysfunction or mutation of the zinc metallopeptidase (ZMPSTE24), which is involved in lamin A post-translational processing, is associated with several metabolic and other pathologies [184,185,186]. The ZMPSTE24 overexpression reduced CS induced by TNF-α through inhibition of RelA nuclear translocation [187]. Furthermore, co-culturing NP cells with bone marrow-derived mesenchymal stem cells reduced degeneration and senescence through upregulation of ZMPSTE24 [187]. Co-culturing degenerated human NP cells with partially digested disc notochordal cells decreased CS while increasing cell viability and proliferation leading to improved matrix health [188], suggesting that trophic factors secreted by notochordal cells could be a promising strategy to improve the disc health. These studies could be a precursor for future research on development of cell-based therapy for IDD.

In addition to modulation of signaling molecules locally within the disc, systemic circulatory factors are also found to affect disc aging phenotype. Exposing old mice to young blood suppressed the expression of CS markers p53, p16^INK4a^, and p21 in disc tissue [189]. This study suggests the existence of one or more blood-borne systemic factors regulating CS and degeneration, which once identified may provide a significant therapeutic target.

## 7. Conclusions and Future Perspectives

Rising interest in disc CS research has led to a growing body of evidence that supports the causal role of CS in driving age-associated IDD. Accumulating during natural aging, disc CS can be accelerated by external and internal stressors, including mechanical, inflammatory, genotoxic, oxidative, and metabolic stress. Senescence of disc cells acquires SASP that is reported to lead to breakdown of ECM and structural instability of IVDs. Conversely, emerging research suggests that elimination of SnCs using genetic strategies and senolytic therapies improved disc tissue structure and physical function in rodent models.

Despite the considerable progress made in the research, the exact molecular mechanisms and regulation of disc CS during age-associated IDD progression remain poorly understood. Although many pathways are involved in modulating disc CS, many of the reported findings require further experimental confirmation. Most of the studies discussed above did not rigorously measure CS with a complete set of biomarkers to determine that disc cells established senescence and not merely transiently expressed one or two biomarkers in response to acute stress. Cellular senescence typically requires 10–14 days to establish post-stress exposure while most of the reported disc literature measured CS 1–3 days post treatment. Hence, much of disc CS literature might have reported cellular acute responses to stress rather than true CS. Therefore, future studies are needed to precisely identify the mechanisms behind aging-associated CS and IDD.

Other important questions remain. Although DNA damage underlies the establishment of CS, what drives disc cellular DNA damage and senescence during aging is not known. How disc CS is modulated by the rise of mitochondrial damage, accumulation of ROS, inflammatory cytokines, or deficiency of growth factors is also not known although these cellular events have been linked in cell culture experiments. Disc CS is reported to be influenced by other cellular processes such as autophagy, mitophagy, and ER stress, but the interaction and regulation among these cellular pathways within the disc’s unique physiological microenvironment during aging is not well understood. Answers to these questions are needed to better understand disc CS to achieve the goal of developing effective senotherapies to treat or delay its impact on age-related IDD.

## Figures and Tables

**Table 1:** Non-coding RNAs regulators of cellular senescence in intervertebral disc.

Non-Coding RNAs	Expression in IDD	In Vitro Study Model	In Vivo Study Model	Senescence Markers	Key Findings	Ref.
circERCC2	↓	Human IDD tissue and NP cells + TBHP siRNAs for miR-182-5p and SIRT1	Rat tail puncture model of IDD, intraperitoneal injection of circERCC2 vectors	SA-β-gal staining	circERCC2 targets miR-182-5p/SIRT1 signaling axis to regulate mitophagy and apoptosis, and reduces senescence to alleviate IDD	[131]
miR-182-5p	↑					
LncRNA H19	↑	Human NP cells +H_2_O_2_	-	SA-β-gal staining, Telomerase activity	Regulation of miR-22 expression by H19 to upregulate the Wnt/β-catenin pathway and promote CS and degenerative changes	[118]
LncRNA TRPC7-AS1	↑	Human disc tissues and NP cells from IDD patients, TRPC7-AS1 siRNA, miR-4769−5p mimics and inhibitor, Hepsin overexpression	-	SA-β-gal staining, p21 and p16 expression	lncRNA TRPC7-AS1 inhibits miR-4769-5p which targets Hepsin to upregulate CS, and regulates NP cell viability and ECM synthesis	[133]
miR-4769-5p	↓					
LncRNA NORAD	↓	Human disc tissues and NP cells + TNF-α stimulation	*NORAD* knockout mice	SA-β-gal staining, p53, p21 and p16 expression	WTAP mediated m^6^A modification of the lncRNA NORAD leading to degradation of E2F3 transcript to promote NP cell senescence	[139]
LncRNA HOTAIR	↑	Human disc tissues and NP cells, HOTAIR overexpression	Rat tail puncture model of IDD, intradiscal injection of HOTAIR siRNA	SA-β-gal staining, p53, p21 and p16 expression	AMPK/mTOR/ULK1 pathway mediated autophagy activation to promote CS and apoptosis	[138]
miR-130b-3p	↑	Human disc tissues and NP cells + TBHP stimulation	Rat tail puncture model of IDD, intradiscal injection of mir-130b-3p inhibitor	SA-β-gal staining, p16 expression	Inhibition of autophagy by miR-130b-3p via ATG14 and AMPK to promote CS miR-130b-3p inhibition alleviates IDD in rat	[43]
miR-217	↓	Human disc tissues and NP cells + TNF-α stimulation MSC-EVs	Rat tail puncture model of IDD, tail vein injection of MSC-EV	SA-β-gal staining, p16 expression	miR-217 targets EZH2 and elevates FOXO3 to activate autophagy and inhibits senescence, apoptosis, and degradation of ECM in NP cells, MSC-EVs carrying miR-217 inhibit IDD in vivo.	[137]

Abbreviations: AMPK, AMP-activated protein kinase; ATG, Autophagy related gene; E2F3, E2F transcription factor 3; EZH2, Enhancer of zeste homolog 2; FOXO3, Forkhead box O-3; H_2_O_2_, hydrogen peroxide; IDD, intervertebral disc degeneration; MSC-EV, Extracellular vesicles from mesenchymal stem cells; mTOR; mammalian target of rapamycin; NP, nucleus pulposus; SA-β-gal, senescence-associated β-galactosidase; SIRT1, Sirtuin 1; TBHP, Tert-butyl hydroperoxide; TNF-α, Tumor necrosis factor alpha; ULK, Unc-51 like autophagy activating kinase; WTAP, Wilms’ tumor 1-associating protein; ↑, increase/activation; ↓, decrease/inhibition; -, not reported.

**Table 2:** Potential therapeutics against cellular senescence in intervertebral disc.

Senotherapy	In Vivo Study Model	In Vitro Study Model	Key Findings	Ref.
Senolytics				
Dasatinib+ Quercitin	C57BL/6 mice aged 6, 14, 18 and 23 months, Weekly i.p. injection of vehicle or 5 mg/kg Dasatinib + 50 mg/kg Quercetin up until 23 months.	-	Inhibition of disc p16, p19, p21 Prevention of age-associated systemic increase in pro-inflammatory mediators and Th-17 related proteins Reduction in disc ECM degradation and NP fibrosis	[161]
Dasatinib+ Quercitin	*Ercc1^−/Δ^* mice, Weekly oral treatment with 5 mg/kg Dasatinib and 50 mg/kg Quercetin or vehicle up to 10–12 weeks.	-	Reduction in physical signs of aging Increased glycosaminoglycan expression in NP tissue of male but not of female mice	[160]
Quercitin	Puncture-induced rat IDD model, Intragastric administration of vehicle (every day) or 100 mg/kg of Quercetin (every other day) for 4 weeks	human NP cells stimulated with 0, 10, and 20 μM IL-1β	Induction of Nrf2 expression leading to inhibition of NF-κB pathway	[52]
Genetic interventions				
*p16^INK4a^*	Young (6 months) and old (22 months) C57BL/6 mice, p16-3MR transgenic mice ± Ganciclovir	-	Increased level of disc p53, p21 and p16^Ink4a^ in old mice, Clearance of SnCs by glaciclovir leading to improved disc health	[1]
*p16^INK4a^*	*Cdkn2a* (p16) knockout mice—mouse tail suspension-induced IVDD model	Human NP cells stimulated with 10 mg/mL IL-1β, 50 nM rapamycin, and p16 siRNA transfection	Reduction in NF-κB activation, ROS levels, SASPs in p16 deficient models Inhibition of p16 by rapamycin	[41]
*p16^INK4a^*	*INK-ATTAC transgenic BubR1*^H/H^ mouse model	-	Reduction in p16^Ink4a^-positive SnCs via FKBP-Casp8 activation Prevention of adipose tissue and muscle loss Delay of age-related phenotypes	[156]
Small biologic drugs inhibiting disc CS				
Curcumin and o-vanillin	-	human NP cells cultured with 5 μM curcumin, 100 μM o-Vanillin, or vehicle (DMSO) for 1 h and 6 h	Reduction in Nrf2 expression via inhibition of NF- κB pathway Decreased expression of p16^INK4a^	[166]
o-vanillin	-	human NP cells treated with 100 μM o-vanillin or vehicle 0.01% DMSO for 4 days then cultured for 21 days	Reduction in TLR-2 expression Reduction in p16, IL-1β, IL-8, NGF, IL-6, and TNF-α expression	[59]
RG-7112 and o-Vanillin	-	human NP cells treated with DMSO vehicle, 100 μM o-Vanillin, or 5 μM RG-7112 for 6 h	Decrease of pro-inflammatory factors INF-γ, IL-6, IL-8, CCL24, and cytokines Reduction in SnCs	[167]
Morroniside	C57BL/6 mice aged 8 weeks Weekly i.p. injection of 20 and 100 mg/kg morroniside up to 8 weeks	rat NP cells treated with 200 and 400 μM morroniside for 2 h before 200 μM H_2_O_2_ exposure	Inhibition of ROS-Hippo-p53 and p21 Reduction in p53 and p21 expression	[162]
Honokiol	Puncture-induced rat IDD model, Oral administration of 40 mg/kg honokiol or 0.5% CMC-Na for 1 week	rat NP cells treated with 0.1–20 μM honokiol for 24 h	Activation of SIRT3 leading to suppression of apoptosis via AMPK-PGC-1α-SIRT3 pathway	[45]
Resveratrol	-	rat NP cells treated with 20 μM resveratrol prior to 100 μM H_2_O_2_ exposure	Activation of SIRT1 via Akt-FoxO1-SIRT1 pathway Reduction in pro-inflammatory cytokines TNF-α, IL-1β, IL-6, IL-8 Reduction in p53, p16, p21	[101]
Metformin	Acupuncture-induced rat IVDD model, i.p. injection of 50 mg/kg metformin for 4 weeks	rat NP cells treated with 10, 50, 100, and 200 μM metformin for 24 h prior to 100 μM TBHP	Inhibition of p16^INK4a^, p53, and p21 via autophagy activation and cGAS-STING-NF- κB pathway inactivation	[168]
Rapamycin	-	rabbit AF cells treated with 50 μg/mL bleomycin + 25 nM rapamycin or 50 μg/mL bleomycin + 50 nM rapamycin for 6 days	Reduction in p16 and p21 expression with rapamycin Reduction in pro-inflammatory factors TNF-α, IL-1β, IL-6, IL-8	[33]
Other interventions				
Omentin-1	-	human NP cells treated with 150 or 300 ng/mL omentin-1 and 10 ng/mL IL-1β for 24 h	Prevention of IL-1β-induced senescence via SIRT1 activation Decreased p16 and p53 expression	[169]
E2	-	rat NP cells treated with 10 ng/mL TNF-α + 10^−7^ M E2 for 24 h and 48 h	Inhibition of ROS/NF- κB activity Increased telomerase activity Reduction in p53 and p16 expression	[170]
Parathyroid hormone	-	rat NP cells treated with 1, 25, and 50 μg/mL dexamethasone for prior to 10 nM PTH for 48 h	Autophagy activation via inhibition of mTOR pathway	[171]
Spermidine	Natural IDD aging mouse model, Daily oral treatment of 25 mg/kg spermidine	human NP cells treated 50 μM spermidine for 24 h prior to 10 ng/mL IL-1β	Prevention of H_2_O_2_ and ROS accumulation via decreased p16 expression	[172]
SB203580	-	rat NP cells cultured at pH 6.2 for 10 days with SB203580	Increased telomerase activity Reduction in p16 and p53 expression Inhibition of p38 MAPK pathway	[68]
Urolithin A	IDD rat model, Daily oral treatment of 25 mg/kg UA for 4 weeks	rat NP cells treated with 80 μM H_2_O_2_ + 20 μM UA	Reduction in oxidative stress via activation of SIRT1/PGC-1α pathway Reduction of p16 and p21 expression	[173]
NAC	Bmi-1^−/−^ mouse Oral treatment of 1 mg/mL NAC	mouse disc cells treated with 2.5 mmol/L NAC for 7–14 days	Prevention of ECM degradation Reduction in oxidative stress via p16^INK4a^/Rb and p19/p53 pathway	[174]

Abbreviation: AF, annulus fibrosus; Bmi-1, B-lymphoma Moloney murine leukemia virus integration site 1; CS, Cellular senescence; ECM, extracellular matrix; H_2_O_2_, hydrogen peroxide; IDD/IVDD, intervertebral disc degeneration; IL, interleukin; NAC, N-acetylcysteine; NF-κB, nuclear factor kappa-light-chain-enhancer of activated B cells; NP, nucleus pulposus; Nrf2, nuclear factor erythroid2-related factor 2; PTH, parathyroid hormone; ROS, reactive oxygen species; SASP, senescence-associated secretory phenotype; SIRT, sirtuin; TNF-α, tumor necrosis factor α; UA, urolithin A; -, not reported.

## Data Availability

Not applicable.
